# Solitary bone plasmacytoma of the axis, a rare and challenging case with good outcomes

**DOI:** 10.1016/j.ijscr.2023.109176

**Published:** 2023-12-20

**Authors:** Ahmed Zendeoui, Mouadh Nefiss, Anis Bousrih, Anis Tborbi, Ramzi Bouzidi, Khelil Ezzaouia

**Affiliations:** Orthopaedic department, Mongi Slim Hospital, Tunisia

**Keywords:** Plasmocytoma, Cervical spine, Case report

## Abstract

**Introduction:**

Plasmacytoma is a rare hematological malignancy with a more favorable prognosis than multiple myeloma. This case report focuses on a unique solitary bone plasmacytoma (SBP) at the craniovertebral junction (CVJ), managed through surgery and radiotherapy.

**Case presentation:**

A 50-year-old patient presented with four months of worsening neck pain and cervicobrachial neuralgia. Despite treatment and physiotherapy, symptoms persisted. Clinical examination revealed neck stiffness, with no motor or sensory deficits. Radiographs and MRI showed an infiltrating axis lesion without instability signs. A CT-guided biopsy yielded inconclusive results. To address instability and establish a diagnosis, a curettage biopsy of the C2 posterior arch was performed, followed by occipito-C4 fusion using an iliac crest graft. Histological examination confirmed SBP. Adjuvant radiotherapy and chemotherapy were administered. At four-year follow-up, there was no multiple myeloma progression, but limited neck mobility were reported, with stable fusion observed on imaging.

**Discussion:**

Solitary bone plasmacytoma primarily affects the axial skeleton, with rare upper cervical spine involvement. Diagnostic criteria include histological confirmation, normal bone marrow analysis, unremarkable imaging (except for the primary lesion), and absence of end-organ damage related to lymphoplasmacytic proliferative disorders. Clinical presentation is nonspecific, and MRI is valuable for soft tissue assessment. Radiotherapy is the primary treatment, with surgery reserved for specific indications.

**Conclusion:**

Solitary bone plasmacytoma is a rare condition with a favorable prognosis when promptly managed. This case underscores the importance of early diagnosis and treatment to prevent recurrence or multiple myeloma progression. A multidisciplinary approach, including surgery when necessary, is crucial for optimal outcomes.

## Highlights

• Plasmacytoma, rare hematological tumor, often better prognosis than myeloma.
• Early diagnosis essential to prevent multiple myeloma progression.
• Solitary bone plasmocytoma diagnosis: histology, marrow, imaging, no end-organ damage.
• Timely management, radiotherapy with surgery if needed, crucial to prevent recurrence.
• Multidisciplinary approach, including surgery when necessary, yields optimal outcomes.

## 1 Introduction

Plasmacytoma is a rare hematological malignant tumor accounting for 5 % of all plasma cell neoplasms. It generally carries a better prognosis compared to multiple myeloma with an average survival rate exceeding 10 years [1,2]. However, there is significant risk of recurrence or myelomatous transformation if not treated in time. Therapeutic management of plasmacytoma is based firstly on radiotherapy. The role of surgery remains a controversial subject.

We report a case of a solitary bone plasmocytoma (SBP) of the craniovertebral junction (CVJ) managed with surgery and radiotherapy. We highlight, clinical presentation, diagnosis criteria, therapeutic strategy and prognosis factors according to recent literature.

This case report has been reported in line with the SCARE Criteria [3].

## 2 Case presentation

We report the case of a 50 years old patient with no previous pathological history, who consulted for neck pain and bilateral cervicobrachial neuralgia evolving for 4 months, progressively worsening and becoming associated with an important functional disability. Symptomatic treatment as well as physiotherapy failed to release symptoms.

Clinical examination showed a slight paravertebral muscles contracture with neck stiffness. No motor or sensory deficit were observed.

**Fig. 1:**
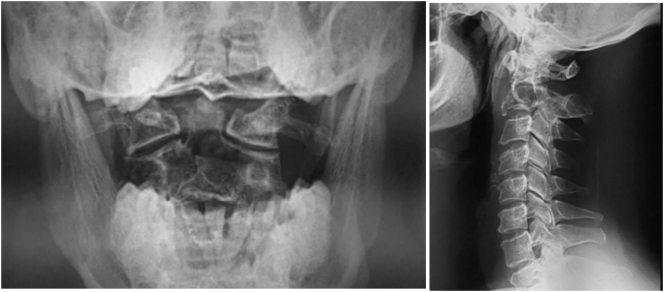
A lateral cervical spine radiograph with an open mouth view shows osteolysis of the odontoid process of the axis without signs of occipitocervical instability.

Radiographs showed a lytic image of the axis without basilar impression or signs of occipitocervical junction instability (Fig. 1).

**Fig. 2:**
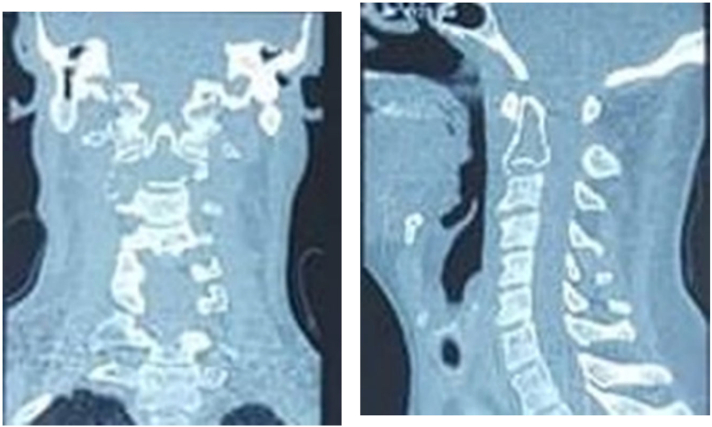
CT scan appearance of a lytic lesion in the axis without detectable instability at the occipitocervical junction.

CT scan was performed showing a lytic image of the axis without signs of occipitocervical junction instability (Fig. 2); The scan-guided biopsy performed by the radiology team involved taking 3 samples containing soft tissue and bone for histological examination. Despite this, the anatomopathological result was inconclusive, and surgical biopsy curettage was deemed necessary to progress in the diagnostic and therapeutic management.

**Fig. 3:**
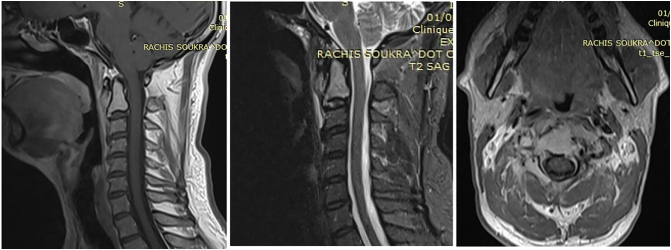
MRI images showing an infiltrative process involving the axis vertebral body, as well as the odontoid process, with invasion of the right side of the posterior arch and extension into the soft tissues.

MRI showed an infiltrating process of the whole vertebral body and odontoid process of the axis with invasion of its right sided posterior arch and soft tissue. (Fig. 3).

**Fig. 4:**
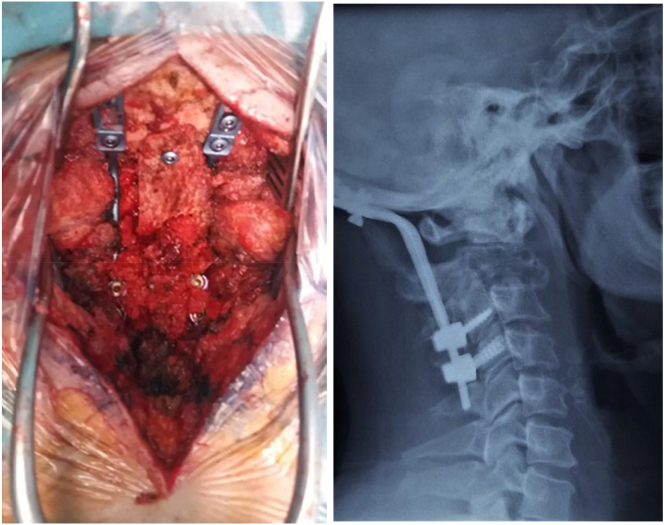
Intraoperative appearance and postoperative imaging following curettage biopsy of the posterior arch of C2 with instrumented occipito-C4 fusion using a posterior iliac graft.

Considering that the bone lesion may potentially lead to occipitocervical instability, we opted for a curettage biopsy of the posterior arch of C2 with occipito-C4 instrumented fusion using posterior iliac crest graft. (Fig. 4).

The diagnosis of SBP was established based on the findings of histological examination and multiple myeloma was excluded based on the results of laboratory tests.

Postoperatively, an adjuvant radiotherapy protocol was prescribed with a dose of 45 Gy as well as a chemotherapy. The evolution was favorable at 4 years fellow-up without progression to multiple myeloma. However, he complained from limitation of neck mobility and limited opening of the mouth.

**Fig. 5:**
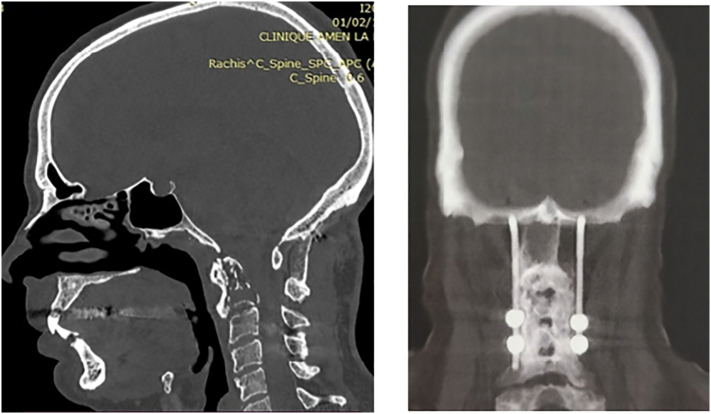
Radiological assessment at the latest follow-up demonstrates a solid fusion without signs of tumor recurrence.

Imaging assessment at the last follow up showed a solid arthrodesis without signs of local tumoral recurrence (Fig. 5).

## 3 Discussion

Solitary bone plasmacytoma primarily affects the axial skeleton and the skull [1]. Among spine involvement, the thoracic spine is the most commonly affected location [4]. The upper cervical spine is rarely involved and represent a more challenging condition due to the risk of CVJ instability [5].

To establish a diagnosis of SBP, the International Myeloma Working Group (IMWG) has defined four essential criteria:

1. Histological confirmation of a single lesion either in bone or soft tissue, with clear evidence of clonal plasma cells.
2. Normal bone marrow analysis without any indications of clonal plasma cells.
3. Normal results of imaging studies (including a skeletal survey and MRI (or CT) scans of the spine and pelvis, except for the primary solitary lesion.
4. Absence of end-organ damage, such as hypercalcemia, renal insufficiency, anemia, or bone lesions (CRAB), that can be attributed to a lymphoplasmacytic proliferative disorder [7,9].

The use of flow cytometry has allowed the IMWG in recent years to revise the diagnostic criteria of SBP and to predict the risk of evolution to MM. In case of positive flow cytometry there is a high risk of progression and the patient should be closely monitored [8,9].

The presence of anemia has been suggested by certain authors as a biological diagnostic factor favoring the diagnosis of multiple myeloma, as over 85 % of multiple myeloma cases are accompanied by anemia [2]. Additionally, when establishing the diagnosis, it is important to include the evaluation of Bence Jones proteinuria, in conjunction with protein electrophoresis, even though monoclonal gammopathy has been detected in nearly half of the reported cases of solitary plasmacytoma [10].

The epidemiological profile of SBP showed a higher incidence in males and an onset age that tends to be about 10 years younger than multiple myeloma. These differences in age and gender distribution between solitary plasmacytoma and multiple myeloma are valuable diagnostic indicators. Additionally, patients with SBP generally have a longer survival rate compared to those with multiple myeloma, and there is a greater likelihood of slower disease progression [11,12].

Clinical presentation of SBP is nonspecific. Axial pain, neuralgia are the most described symptoms. Pathological fractures can reveal the diagnosis in its advanced stage [10,13].

Radiograph often yield negative results when dealing with, as approximately 25 % to 50 % of bone trabeculae must be damaged before a visible bone defect appears [13]. To address this limitation, Chua et al. have demonstrated the potential of PET scans in cases where there is a strong clinical suspicion despite negative radiological findings, offering a promising avenue for disease recurrence detection [4].

Magnetic Resonance Imaging (MRI) is a highly effective tool for evaluating medullary invasion and soft tissue extension. It typically reveals bone lesions characterized by T1 isosignal and T2 hypersignal, which become more pronounced following the administration of contrast material [14].

Radiotherapy stands as the primary treatment approach for SBP, either as a standalone treatment or in conjunction with surgery. When administered at optimal doses ranging from 40 to 50 Gy, it achieves a tumor control rate exceeding 90 %, characterized by excellent patient tolerance and a rapid and long-lasting analgesic effect [15]. In cases involving vertebral lesions, treatment fields typically include one to two adjacent vertebrae above and below the affected level [14].

The role of surgery in the management of bone plasmacytoma is a subject of controversy in the literature. In a multivariate study conducted by Kilciksiz et al. with a follow-up period of 2.4 years, superior outcomes were observed in patients who received combined surgery and radiotherapy compared to those solely treated with high doses of radiotherapy. Furthermore, the combined therapy approach is recommended when complete surgical tumor removal is not feasible [14].

While complete surgical resection may be considered for accessible peripheral lesions, it should be undertaken with caution in spinal localization given the therapeutic effectiveness of radiotherapy and to avoid the risk of complications related to some unnecessary surgery [13,15]. However, surgery often remains necessary in cases of spinal cord compression or spinal instability.

In our specific case, the main objective of surgery was to establish the right diagnosis given the inconclusive result of the CT-guided biopsy and to stabilize the CVJ by providing biomechanical support to the spine. Transoral approach of the axis to remove the entire tumor was avoided due to the increased risk of subsequent complications and tumor control in this area was left to radiotherapy.

Disease recurrence or spreading typically correlates with the appearance, reappearance, or elevation of monoclonal immunoglobulin levels in the patient's serum or urine. This underscores the significance of conducting immunochemical studies on the serum and urine of patients during long-term follow-up [1]. The progression patterns of solitary plasmacytoma include local recurrence, lymph node infiltration, and transformation into myeloma. On average, disease recurrence occurs approximately two years after initial treatment, and all recurrences, including those mentioned, typically manifest within the initial five years following treatment [13]. The primary indicators for the likelihood of recurrence are the size of the tumor and the radiation dose administered, with a greater risk associated with tumors larger than 5 cm and radiation therapy doses less than or equal to 35 Gy [13,15].

Furthermore, it's important to note that even after years of remission, metastasis remains a possibility in cases of solitary plasmacytoma. These metastases may affect bone or extramedullary sites, including but not limited to the thyroid, lymphoid nodules, and the parotid gland [1,2,16].

The assessment of bone marrow plays a pivotal role in establishing the prognosis of the disease. Prognostic outcomes are contingent upon the identification of clonal plasma cells within the bone marrow. Consequently, two distinct classifications have been delineated: solitary plasmacytoma (where clonal plasma cells are absent) and solitary plasmacytoma with limited marrow involvement (involving less than 10 % clonal cells). Consequently, bone marrow examinations revealing clonal cell infiltration of 10 % or more are categorized as indicative of multiple myeloma.

If left untreated, a solitary bone plasmacytoma has the potential to advance into a disseminated form of multiple myeloma [2,17], and this progression can occur in up to 10 % of cases within a period of three years [9].

## 4 Conclusion

Solitary bone plasmocytoma is an uncommon tumor that tends to predominantly impact the axial skeleton and the skull. Diagnosis is based on a comprehensive evaluation including clinical, histological, biological, and radiological parameters. Effective management is crucial to prevent recurrence and the potential transformation into myeloma. Treatment typically involves radiotherapy, supplemented by surgery when necessary to decompress the spinal cord or stabilize the column.

## Consent statement

Written informed consent was obtained from the patient for publication of this case report and accompanying images. A copy of the written consent is available for review by the Editor-in-Chief of this journal on request.

## Provenance and peer review

Not commissioned, externally peer-reviewed.

## Ethical approval

Ethical approval for this study was provided by the Ethical Committee of Mongi Slim University Hospitals, Marsa, Tunisia on 01 October 2023.

## Funding

This research did not receive any specific grant from funding agencies in the public, commercial, or not-for-profit sectors.

## Author contribution

Ahmed Zendeoui: original draft writing

Mouadh Nefiss: Data analysis

Anis Bousrih: Data collection

Anis Tborbi: Paper editing

Ramzi Bouzidi: Supervision

Khelil Ezzaouia: Paper validation

## Guarantor

Ahmed Zendeoui

## Declaration of competing interest

The author(s) declared no potential conflicts of interest.
